# Host Plant Effects on Alkaline Phosphatase Activity in the Whiteflies, *Bemisia tabaci* Biotype B and *Trialeurodes vaporariorum*

**DOI:** 10.1673/031.011.0109

**Published:** 2011-01-27

**Authors:** Ying Yan, Lu Peng, Wan-Xue Liu, Fang-Hao Wan, Marvin K. Harris

**Affiliations:** ^1^ State Key Laboratory for Biology of Plant Diseases and Insect Pests, Institute of Plant Protection, Chinese Academy of Agricultural Sciences, Beijing 100094, PR China; ^2^ Key Laboratory of Entomology and Pest Control Engineering, College of Plant Protection, Southwest University, Chongqing 400716, PR China; ^3^ Department of Entomology, Texas A&M University of USA, TX, USA

**Keywords:** greenhouse whitefly, invasion, saliva, selerotization, silverleaf whitefly, sucrose metabolism

## Abstract

*Bemisia tabaci* (Gennadius) B-biotype and *Trialeurodes vaporariorum* (Westwood) (Hemiptera: Aleyrodidae) often coexist on greenhouse-grown vegetable crops in northern China. The recent spread of *B. tabaci* B-biotype has largely replaced *T. vaporariorum*, and B-biotype now overlaps with *T. vaporariorum* where common hosts occur in most invaded areas. The impact of the B-biotype on the agro eco system appears to be widespread, and involves the ability to compete with and perhaps replace other phytophages like *T. vaporariorum*. An emerging hypothesis is that the B-biotype is physiologically superior due at least in part to an improved ability to metabolically utilize the alkaline phosphatase pathway. To test this hypothesis, alkaline phosphatase activity was studied in the B-biotype and *T. vaporariorum* after feeding on a number of different hosts for a range of durations, with and without host switching. Alkaline phosphatase activity in *T. vaporariorum* was 1.45 to 2.53-fold higher than that of the B-biotype when fed on tomato for 4 and 24 h, or switched from tomato to cotton and cabbage for the same durations. However, alkaline phosphatase activity in the B-biotype was 1.40 to 3.35-fold higher than that of *T. vaporariorum* when the host switching time was ∼72 and ∼120 h on the same plant. Both short-term (4 h) and long-term (72 h) switching of plant hosts can significantly affect the alkaline phosphatase activity in the two species. After ∼120 h, feeding on tomato and cotton alkaline phosphatase activity in the B-biotype was significantly higher than that of *T. vaporariorum*. It was shown that alkaline phosphatase aids the species feeding on different plant species, and that the B-biotype is physiologically superior to *T. vaporariorum* in utilizing the enzyme compared to *T. vaporariorum* over longer periods of feeding.

## Introduction

The silverleaf whitefly, *Bemisia tabaci* (Gennadius) B-biotype (also named *B. argentifolii*) (Hemiptera: Aleyrodidae) is polyphagous, attacking more than 600 different plant species in both field and greenhouse settings, including food, fiber, and ornamental species (Perring et al. 1993). This so-called “super bug” (Barinaga 1993) is a major crop pest on all continents except Antarctica (Brown et al. 1995). The greenhouse whitefly, *Trialeurodes* *vaporariorum* (Westwood) is also a serious polyphagous pest of greenhouse plants. World-wide these species cause billions of dollars in losses each year by feeding on plant phloem, transmitting plant pathogenic viruses, and producing a sticky honeydew that supports the growth of sooty mold (Zalom et al. 1995; Liang et al. 2007; Jiu et al. 2007). Moreover, the B-biotype was listed in the top 100 invasive species (International Union for the Conservation of Nature and Natural Resources) and has displaced non B-biotype whitefly in the USA (Perring et al. 1993), Mexico (Costa et al. 1993a), Colombia (Quintero et al. 1998), Australia (De Barro and Hart 2000), south China (Liu et al. 2007), and Brazil (Lima et al. 2002). In north China, *T. vaporariorum* was the predominant whitefly species in the greenhouse from the mid-1970s to the mid-1990s (Luo et al. 2000). However, since the invasion of China by the B-biotype, densities of *T. vaporariorum* have declined considerably (Cui et al. 2008).

Some detoxification and digestive enzymes have been previously studied to explore why the B-biotype was displacing *T. vaporariorum* (Lei et al. 2006; Liang et al. 2007; Zhang et al. 2008; An et al. 2008). The hypothesis was that these enzymes had greater activity in the B-biotype than they did in *T. vaporariorum*. Alkaline phosphatase (ALP: EC 3.1.3.1) is a ubiquitous enzyme in all organisms and is used to hydrolyze orthophosphate monoesters; their precise physiological function remains unclear, but they are considered to play a role in phosphate uptake and in secretory processes in epithelia in mammals. In insects, functional ALPs was found in *Leptinotarsa decemlineata* (Yi and Adams 2001), *Bombyx mori* gut (Eguchi et al. 1990), in *Drosophila melanogaster* brain and malpighian tubules (Yang et al. 2000; Cabrero et al. 2004), and in several mosquito species (Igbokwe and Mills 1982; Houk and Hardy 1984). It has previously been reported that in the B-biotype, ALP was located in the saliva, salivary gland, and some other tissues (Funk 2001). The role of ALP is again unclear, but it has been suggested that salivary ALP aids in plant feeding, while the ALP in other tissues may have a role in cuticle development and metabolism. The kinetic properties of ALP from different developmental stages of the Bbiotype and *T. vaporariorum* indicated that ALP may be involved in their relative competitiveness (Yan et al. 2008). Additionally, although the B-biotype and *T. vaporariorum* occupy virtually the same ecological niche and coexist on many plants, their overall host range and host preference appear to be different and this may play an important role in their competition (Lei et al. 2006; De Barro et al. 2006; Zhang et al. 2008). In this study, four host plants: tomato (equally suited to both species; used as the control), cotton (more suitable to the Bbiotype than to *T. vaporariorum*), cabbage (suitable for the B-biotype, but unsuitable for *T. vaporariorum*), and celery (suitable for *T. vaporariorum*, but unsuitable for the Bbiotype) were chosen in this experiment (Lei et al. 2006; Luo et al. 2007; Zhang et al. 2008).

## Materials and Methods

### Chemicals

2-(N-morpholino) ethanesulfonic acid (MES), N-tris (hydro xymethyl) methyl-3-aminopropanesulfonic acid (TAPS) were purchased from Sigma (www.sigmaaldrich.com), N-2-hydroxyethylpiperazine-N′-2-ethanesulfonic acid (HEPES), N-tris (hydroxymethyl) methyl-3-aminopropanesulfonic acid (TAPS), 2-N- cyclohexylamino ethanesulfonic acid (CHES), 3-(cyclohexy lamino)-1-propanesulfonic acid (CAPS), Purified bovine serum albumin (BSA), and *p*-nitrophenyl phosphate (pNPP) were obtained from Amresco (www.amresco.com).

### Insects and host plants

The B-biotype and *T. vaporariorum* were originally collected from tomato plants, *Lycopersicon lycopersicum* L. (Solanales: Solanaceae), in the experimental fields of the Chinese Academy of Agricultural Sciences, Beijing, in 2003. Colonies of the B-biotype were maintained on tomato (3 years), cotton, *Gossypium hirsutum* (L.) (Variety ‘Simian-3’) (4 years), and cabbage, *Brassica oleracea* var. capitata L. (JingFeng-1) (1 year). *Trialeurodes vaporariorum* were maintained on tomato (3 years), cotton (4 years), and celery, *Apium graveolens* L. (ZhongQin-2) (1 year) in separate greenhouses at 20–34° C, 50–60% RH and natural photoperiod (39° 55′ N, 116° 20′ E). The five-to-six true leaf stage of tomato, seven-to-eight true leaf stage of cotton, five-to-seven true leaf stage of cabbage, and six-to-eight true leaf stage of celery were used in this experiment, and no plants had flowers or flower buds. All of the plants were individually grown in plastic pots (9 cm in diameter) in greenhouses and no chemicals were sprayed onto the plants during the growth and test period.

### Saliva collection

Up to 200 B-biotype and *T. vaporariorum* adults were transferred from plants to a feeding chamber, a standing cylinder (glass tube) measuring 30 mm in diameter and 80 mm high and covered on top by a double layer of Parafilm™ with one of the diet packets in between (a “sachet”).

Each sachet contained 400µl of food (10% sucrose, 100mM MES, pH6.5). B-biotype and *T. vaporariorum* remained in the chamber in a climatic incubator (MHT350, Sanyo Electric Co., Ltd., www.sanyo.com) at 25° C, 60% RH and 14:10 L:D photoperiod for 24 h. Then the sucrose diet that contained salivary secretions was collected from three feeding chambers. The sucrose diet was diluted with MilliQ water (Millipore, www.waters.com) and concentrated to approximately 250µl using Vivaspin (www.vivaproducts.com) 2ml with a 30-kDa MW cut-off. Purified bovine serum albumin was added to the solution in the concentrators (to a final concentration of approximately 0.25 mg/ml); this aided in protein stabilization and prevented nonspecific binding to the concentrator. This solution did not have detectable ALP activity when tested separately (not shown).

### Host plant effect experiments

Tissue testing was divided into two experiments. The first experiment was designed to test the effect of switching host and time on ALP activities of the two whiteflies. Newly emerged adults of the Bbiotype and *T. vaporariorum* (≈5 h) were collected from the tomato and sexed using a stereomicroscope, then transferred to the 1–3 top leaves of the test plants:, tomato (T-T, control), cotton (T-CO), cabbage (T-CA), and celery (T-CE), in clip-cages. Ten pairs of whiteflies were put into each cage. The whiteflies were then collected from the cage after 4 h (timing commenced after the whiteflies had begun to feed), 24 h (when the color of the whiteflies' tissues had begun to darken), 72 h (when ∼80% of the whiteflies had died on the unsuitable host), and 120 h (when all the whiteflies died on the unsuitable host); whiteflies were sexed again before undertaking the enzyme essay. The second experiment was designed to test the ALP activity of the two whiteflies when confined to a single host. B-biotype adults (five days old) from tomato, cotton, and cabbage colonies and *T. vaporariorum* adults (five days old) from tomato, cotton, and celery were collected for use in the experiment.

Mature adult whiteflies were collected from each host and transferred to the feeding chamber for saliva testing. Preliminary testing showed no significant differences in the salivary ALP activity between the male and female, or between newly emerged adults (≈5 h old) and mature adults (≈5 d old), so adults were not standardized for age or sex.

### Alkaline Phosphatase Assays

For tissue test, 5 pairs of freshly collected whiteflies were homogenized at 4° C in a Eppendorf 1.5ml concentrator containing 100µl of 10mM magnesium acetate-sodium acetate solution (pH7.0) The homogenate was centrifuged at 10,000g for 15 min and the supernatant then used in the assays. ALP activity was measured by incubating 40µl of the supernatant at 37° C in a 400µl assay containing 100mM (TAPS), pH 7.8, 0.75mM pNPP as described in Yan et al. (2008). Reactions were terminated after 30 min using an ice-bath. To estimate the degree of pNPP hydrolysis, *p*-nitrophenol (PNP) was quantified with a spectrophotometer at 405nm. The enzyme unit (U/mg protein) was defined as the production of 1 µmol of pnitrophenol by a reaction between 1 mg protein and related substrate per min at 37° C.

The measurement protocols for testing saliva were the same as those for the tissue tests, but the optimal pH for salivary ALP and an aliquot (250µl) of the diet were used. To determine the optimal pH range of salivary ALP, the whiteflies from tomato were used and one of several buffers was added to reach a final concentration of 100mM: HEPES, pH 6.5–7.4; TAPS, pH 7.4–9.4; CHES, pH 9.0– 10.0; CAPS , pH 9.7–11.0. Then ALP activity was measured by incubating an aliquot (40µl) of the concentrated whitefly diet at 37° C in a 200µl reaction system containing the indicated buffer and 0.75mM *p*-nitrophenyl phosphate (pNPP) for 30 min. The activity was determined at 37° C by measuring the increase in absorbance at 405 nm on a micro titer plate spectrophotometer (Bio-RAD, model 680, USA). Protein concentration in whitefly tissue extracts and saliva was determined by the method of Bradford (1976) using bovine serum albumin as the standard.

### Statistical analysis

Statistical analysis was performed using SAS Institute 8.0 Inc. USA (2002). Species differences were analyzed by a two-sample t test for means and the effects of timed transfers between and within plant species on ALP activity of the B-biotype or *T. vaporariorum* were analyzed by one-way ANOVA; Fisher's LSD was used to separate means. A value of *P*≤0.05 was accepted as statistically significant.

## Results

### ALP activity in tissues of the two species transferred between different host plants

**Figure 1.**
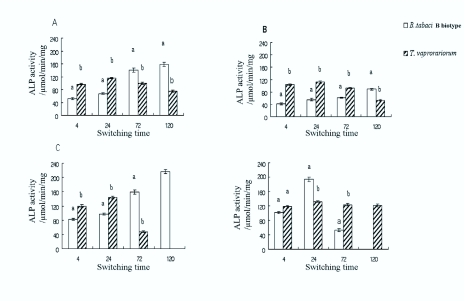
The comparison of ALP activity from *Bemisia tabaci* B-biotype and *Trialeurodes vaporariorum* in different time sets on the control plant (tomato, A), and the transfer plants: cotton (B), cabbage (C), and celery (D). Each bar presents mean ± SE, n = 5. (The bars in the same time set with different letters are significantly different at *p*<0.05, two Sample t test for means). High quality figures are available online.

The ALP activity in the B-biotype increased in the T-T, T-CO, and T-CA tests (Figure 1: A, B, C). However, ALP activity in the T-CE and T-CA tests peaked at 24 h before dropped sharply at 72 h; all B-biotype and *T. vaporariorum* were dead by 120 h after transfer (Figure 1: C, D). The ALP activity in *T. vaporariorum* peaked at 24 h, then decreased in all host combinations (Figure 1: A, B, C, D). In T-CE, *T. vaporariorum* maintained a relatively high and stable ALP activity (Figure 1: D). A significant difference was observed in the ALP activities from both B-biotype and *T. vaporariorum* in different time sets on the plant transfer studies, except at 4 h in the T-CE test (P<0.05).

### Effects of plant species transfers and time on ALP activity in tissues of the two whitefly species

Short-term (4 h) transfer between plant species positively affected the ALP activity in the tissues of B-biotype and *T.vaporariorum*, except in the T-CO test. Compared with the control, the ALP activity increased 57 % (P<0.001) and 24% (P<0.001) in the T-CA test, and increased 93% (P<0.001) and 22% (P<0.001) in the T-CE test of the B-biotype and *T. vaporariorum*, respectively (Table 1). However, long-term (72 h) switching of plant species inhibited the ALP activity in tissues of the two species, except when this occurred on the preferred host. Compared with the control, the ALP activity decreased 56% (P<0.001) and 7%, respectively, in the T-CO test in tissues of the B-biotype and *T. vaporariorum*. In the T-CE test, the ALP activity in the Bbiotype decreased 62% (P<0.001) while that of *T. vaporariorum* maintained 124 % compared with the control treatment. In the T-CA test, the ALP activity in the B-biotype maintained 114 %, while that of *T. vaporariorum* decreased 52% compared with the control (P<0.001, Table 2).

**Table 1.**
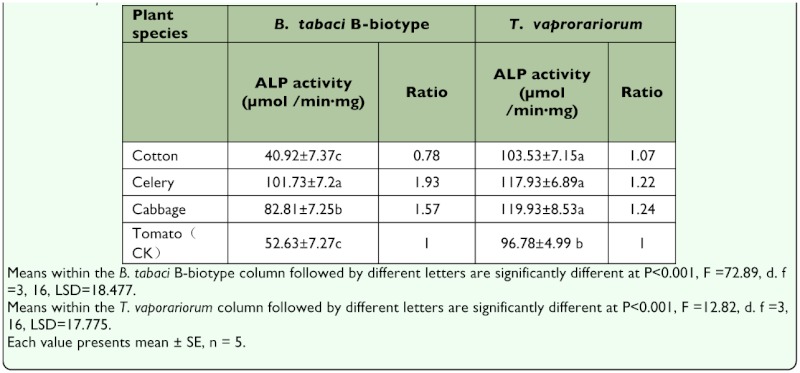
Effects of short-term (4h) switching plant species on the ALP activity in the tissues of *Bemisia tabaci* B-biotype and *Trialeurodes vaporariorum*.

### The pH optimum for salivary ALP

**Figure 2.**
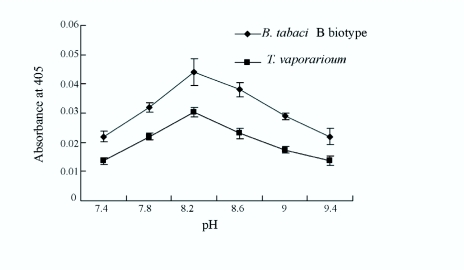
Activity profile of salivary ALP from *Bemisa tabaci* B-biotype and *Trialeurodes vaporariorum* as a function of pH in 100 mM TAPS buffer. Activity was assayed with the chromogenic substrate pNPP and readings were taken on a micro titer plate spectrophotometer (405 nm). Each point represents mean ± SE, n = 3. High quality figures are available online.

The activities of salivary ALP for both Bbiotype and *T. vaporariorum* increased from pH 7.4 and peaked at pH 8.2, then decreased at pH 9.4 indicating that optimal activity was present at pH 8.2. In the 7.4–9.4 pH range, the salivary ALP responses from the Bbiotype were generally higher than those from *T. vaporariorum* (Figure 2). In addition, salivary ALP from both species could be detected at pH 7.0 (the approximate pH of phloem sap) using 100 mM HEPES buffer (not shown).

**Table 2.**
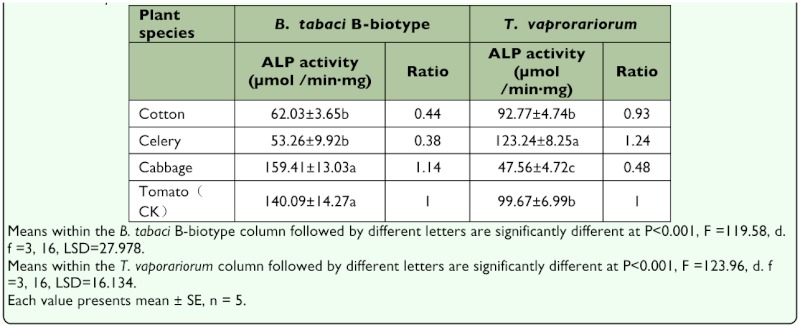
Effects of long-term (72h) plant species switching on ALP activity in tissues of *Bemisio tabaci* B-biotype and Trialeurodes *voporariorum*.

### Effects of plant species on ALP activities in tissues and saliva of the two whitefly species

The ALP activity in the tissue and saliva of the B-biotype from cabbage maintained 144% (P<0.001) and 142% (P<0.001) and those from cotton maintained 31% (P<0.001) and 125% (P<0.001), compared with the control. The ALP activity in the tissue and saliva of *T. vaporariorum* from cotton maintained 60% (P<0.001) and 184% (P<0.001) compared with the control plant. The ALP activities were maintained (significantly) higher in the tissue and saliva of the B-biotype compared with those from *T. vaporariorum* when fed on tomato and cotton (*P* < 0.001, Table 3).

**Table 3.**
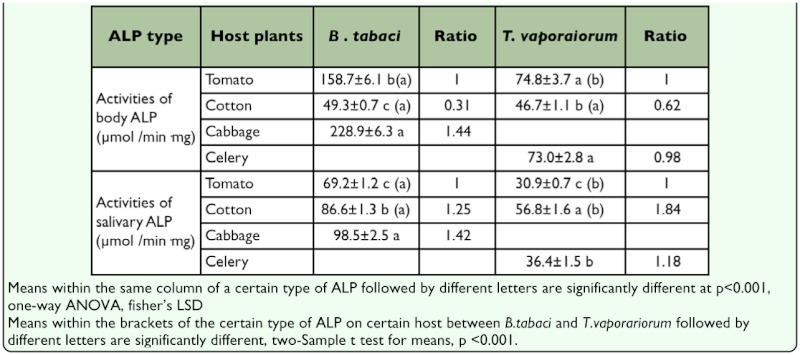
Effects of plant species on ALP activity in the tissues and saliva of *Bemisio tobaci* B-biotype and *Trioieurodes vaporariorum*.

## Discussion

B-biotype and *T. vaporariorum* ALPs were located in the salivary gland and saliva, accessory salivary gland, colleterial gland, gut, and ovariole (Funk 2001). These ALPs appear to have crucial roles in absorption, development, and cuticle formation. Moreover, significantly lower ALP activity in the immature form and higher ALP activity in the adult (3 d) were found in tissues of the Bbiotype compared with *T. vaporariorum* on tomato (Yan et al. 2008). The results in this study showed that significantly lower ALP activity was still maintained in tissues of *B. tabaci* B-biotype at 4 and 24 h after eclosion compared with *T. vaporariorum* in the T-T and T-CO tests. During this period, the tissues of newly emerged B-biotype and *T. vaporariorum* become opaque, a process that is accompanied by intensive sclerotization. Lower ALP activity just after eclosion may account for the smaller tissue and body size of the B-biotype compared with *T. vaporariorum* (Hu and Wu 2001). It was considered that whiteflies may be comparable to *D. melanogaster* in using ALP. In *D. melanogaster*, tyrosine is stored in hemolymph as tyrosine-O-phosphate. During cuticle formation, ALP hydrolyzes the phosphate moiety and makes tyrosine available for conversion to catecholamines that are then used to cross-link proteins during sclerotization (Lunan and Mitchell 1969). Evidence for this role in B-biotype and *T. vaporariorum* will require further investigation, including determining whether soluble tyrosine-O-phosphate is present in these whiteflies, as it is in *Drosophila* (Funk 2001).

It was shown that after adult B-biotype and *T. vaporariorum* entered a relatively stable developmental and feeding stage, the ALP activity in tissues of the B-biotype was observed to increase and exceed those of *T. vaporariorum* at 72 h (T-T) and 120 h (T-CO) after host switching, which indicated that the ALPs (may be different isozymes) were produced to aid in absorption and substance metabolism. Generally, insect gut ALPs play a role in epithelial transport (Eguchi 1995; Yan et al. 2009). For example, two silkworm gut isozymes, a membrane-bound form (m-ALP) and a soluble form (s-ALP), are believed to participate in the transport of glucose and fatty acids across intestinal wall membranes (Srivastava and Bhat, 1963). Furthermore, mALP is thought to be involved with digestion and absorption of nutrients by the columnar epithelial cells. However, s-ALP is present in goblet cells and is involved in the regulation of ionic balance (Eguchi et al. 1990; Eguchi 1995). The patterns of s- or m-ALP-specific activities were identified in the beetle midgut and were correlated with feeding activity of Colorado potato beetle, *Leptinotarsa decemlineata* (Yi and Adams 2001). Several studies have also reported that mammal and insect ALPs were correlated to sucrose metabolism (Crane 1960; Dimitriadis and Kastritsis 1985; Jimenez and Gilliam 1990; Yamamoto et al. 1991). The B-biotype and *T. vaporariorum* are piercing-sucking insects with stylet-like mouthparts that are used to feed on the phloem sap, which mainly contains sucrose. The ALP activity in tissues of the B-biotype increased in the T-T, T-CO, and T-CA tests which may represent an adaptation to the plant nutrients. In contrast, the ALP activity in tissues of *T. vaporariorum* dropped sharply which may indicate a low utilization of sucrose. Considering *T. vaporariorum* was not as competitive as the B-biotype on tomato, cotton, and cabbage, it was hypothesized that although the Bbiotype′s use of ALP to promote sclerotization and development during the juvenile period may not be as good as that of *T. vaporariorum*, the ability of the adult to utilize the enzyme to absorb and metabolize was markedly stronger than that of the *T. vaporariorum* adult.

As polyphagous insects, B-biotype and *T. vaporariorum* can adjust their digestive enzymes to adapt to changing nutrient conditions of their host plant(s) in a very short time (Lei et al. 2006; Zhang et al. 2008). *Bemisia tabaci* begins to metabolize sucrose just 3 h after being transferred from cotton to pumpkin (Gerling and Lindenbaum 1991). It was confirmed that the ALPs in tissues of these two species were also significantly affected 4 h after host switching. The ALP activities in tissues of both species were activated and maintained at a relatively high level after they were switched to the preferred host; they were initially actively expressed on the preferred host and then suppressed after transfer to an unsuitable host. Insects benefit from ALP and low titers are harmful to the insect's well being, which explains why some insecticides target ALPs (Tripathi 1982; Misbahuddin and Ehteshamuddin 2000). Furthermore, some plant secondary substances are able to inhibit ALP and so reduce food utilization (Nathan 2006; Nathan et al. 2007). It can therefore be inferred that activated ALP can help B-biotype and *T. vaporariorum* feed on their preferred host, while inhibition of ALP will reduce the effective use of food sources and result in death on unsuitable hosts. In contrast, the ALP activities in tissues of B-biotype and *T. vaporariorum* were also inhibited in the T-CO test, but both species performed well on cotton. It was reported that when the B-biotype and *T. vaporariorum* were transferred from tomato to cotton, cabbage, and maize the contents of trehalose from both species decreased significantly on cotton only (Lei et al. 2006). Since sucrose is the precursor to trehalose production in B-biotype and *T. vaporariorum*, it was assumed that there a correlation exists between inhibited ALP and low contents of trehalose in both species on cotton.

It was stated that changes in diet were reflected in salivary composition of various Heteroptera and Homoptera (Miles 1987). Also, it was found that different host plants can alter hemipteran salivary protein profiles (Habibi et al. 2001). It was confirmed that different host species can significantly affect the ALP activities in the tissues and saliva of B-biotype and *T. vaporariorum*. Generally, *B. tabaci* B-biotype maintained higher ALP activities in tissues and saliva than *T. vaporariorum* from different host colonies. Moreover, significantly higher ALP activities were found in the tissues and saliva of the Bbiotype in cabbage compared with those in tomato (144% and 142%, respectively). Again, this may indicate that a high level of ALP can help the B-biotype to be more competitive than *T. vaporariorum*. Interestingly, like the trend in the host transfer tests, cotton plants negatively affected ALP activities in the tissues of both species (31% and 62%, P<0.001), but both species from cotton maintained a significantly higher salivary ALP activity (125% and 184%, P<0.001) compared with those from tomato. It was reported that B-biotype stress can result in sucrose accumulation in cotton leaves (Lin et al. 2000). In plant cells, sucrose is synthesized *de novo* from uridine 5′diphosphoglucose (UDPGIc) and fructose-6-phosphate (Fru-6-P) in a sequence of two reactions catalyzed by sucrose phosphate synthase (SPS, EC 2.4.1.14) and sucrose-phosphate phosphatase (SPP, EC 3.1.3.24). Since SPP and ALP are closely related and share a similar function (to hydrolyze phosphate esters), it was hypothesized that salivary ALP, which is injected into plant tissue by whiteflies, may be used to generate more food, i.e. sucrose. B-biotype may use ALP to interfere with plant photosynthesis and thereby modify the food source to benefit themselves. Salivary components of *B. tabaci* B-biotype nymphs (Costa et al. 1993b; van de Ven 2000) and adult males (De Barro and Khan, 2007) may induce plant damage, such as tomato irregular ripening and squash silverleaf syndrome. However, B-biotype and *T. vaporariorum* salivary ALP may not induce such systemic responses because *T. vaporariorum* adults, which are capable of ALP injection, do not induce these symptoms.

In north China, the B-biotype has largely replaced *T. vaporariorum* (Lin et al. 2007). Whiteflies disperse from the greenhouse to the field crop in the spring and frequently switch among host plants during summer and fall. We have shown that ALP activities in tissues and saliva of these two whitefly species were involved in sucrose metabolism, which can help whiteflies feed on different plants. In addition, the B-biotype showed a greater ability than *T. vaporariorum* to utilize ALP, which may help us understand the mechanisms aiding invasion, host adaptation and competition among whiteflies.

## Abbreviationos

**ALP**,: alkaline phosphatase
